# Intravenous Lidocaine as an Adjunct for Postoperative Recovery After Open Abdominal Surgery: A Systematic Review

**DOI:** 10.3390/jcm15114068

**Published:** 2026-05-25

**Authors:** Calin Muntean, Melania Veronica Ardelean, Vasile Gaborean, Ionut Flaviu Faur, Alaviana Monique Faur, Razvan Constantin Vonica, Catalin Vladut Ionut Feier

**Affiliations:** 1 Department III-Functional Sciences, Medical Informatics and Biostatistics, “Victor Babes” University of Medicine and PharmacyTimişoara, Eftimie Murgu Sq. No. 2, 300041 Timişoara, Romania; cmuntean@umft.ro; 2 Department V, Internal Medicine I-Discipline of Medical Semiology I, “Victor Babes” University of Medicine and Pharmacy Timişoara, Eftimie Murgu Sq. No 2, 300041 Timişoara, Romania; 3 Center of Advanced Research in Cardiology and Hemostasology, “Victor Babes” University of Medicine and Pharmacy, Eftimie Murgu Sq. No 2, 300041 Timişoara, Romania; 4 Thoracic Surgery Research Center, “Victor Babes” University of Medicine and Pharmacy Timişoara, Eftimie Murgu Sq. No. 2, 300041 Timişoara, Romania; 5 Department of Surgical Semiology, Faculty of Medicine, “Victor Babes” University of Medicine and Pharmacy Timişoara, Eftimie Murgu Sq. No. 2, 300041 Timişoara, Romania; 6 IInd Surgery Clinic, Timisoara Emergency County Hospital, 300723 Timişoara, Romania; flaviu.faur@umft.ro; 7 X Department of General Surgery, “Victor Babes” University of Medicine and Pharmacy Timişoara, 300041 Timişoara, Romania; 8 Department of Doctoral Studies, “Victor Babes” University of Medicine and Pharmacy Timişoara, Eftimie Murgu Sq. No. 2, 300041 Timişoara, Romania; 9 Preclinical Department, Discipline of Physiology, Faculty of Medicine, “Lucian Blaga” University of Sibiu, 550169 Sibiu, Romania; razvanconstantin.vonica@ulbsibiu.ro; 10 Abdominal Surgery and Phlebology Research Center, “Victor Babes” University of Medicine and Pharmacy, 300041 Timişoara, Romania; catalin.feier@umft.ro; 11 First Surgery Clinic, “Pius Brinzeu” Clinical Emergency Hospital, 300723 Timişoara, Romania

**Keywords:** intravenous lidocaine, lignocaine, open abdominal surgery, postoperative pain, opioid-sparing analgesia, postoperative ileus, enhanced recovery after surgery, PRISMA, systematic review, randomized controlled trial

## Abstract

**Background/Objectives:** major open abdominal surgery remains associated with clinically important postoperative pain, delayed gastrointestinal recovery, opioid exposure, and prolonged length of stay. Intravenous lidocaine infusion (IVLI) has biologically plausible analgesic, anti-hyperalgesic, anti-inflammatory, and opioid-sparing effects, but prior evidence syntheses have often combined open and minimally invasive procedures. This systematic review evaluated evidence for perioperative IVLI in adult patients undergoing major open abdominal surgery. **Methods:** the review was structured according to PRISMA 2020. The final search was run on 15 January 2026 and covered PubMed/MEDLINE, Embase, Cochrane CENTRAL, Scopus, Web of Science Core Collection, ClinicalTrials.gov, and WHO ICTRP from database inception to that date, without language restrictions at the search stage. Eligible studies enrolled adults undergoing elective open abdominal surgery and compared systemic IVLI with placebo, usual care, or active epidural analgesic comparators. Primary outcomes were postoperative opioid consumption and pain intensity. Secondary outcomes included gastrointestinal recovery, postoperative ileus, length of hospital stay, postoperative nausea and vomiting, inflammatory/stress biomarkers, and adverse events. **Results:** ten randomized trials involving 658 participants were included. Placebo/usual-care trials and active-comparator trials were synthesized separately because they address different clinical questions. IVLI generally reduced opioid consumption compared with placebo, with extractable effects including a 55.9 mg reduction in 72 h morphine use in one abdominal surgery trial and a 13.9 mg reduction in 24 h morphine use after radical prostatectomy. Gastrointestinal recovery favored IVLI in most placebo-controlled studies; for example, first flatus occurred 12.5 h earlier and first bowel movement 28.4 h earlier in one trial. Active-comparator trials suggested comparable early dynamic pain outcomes versus thoracic epidural analgesia in selected settings, although opioid consumption findings were less consistent. No serious lidocaine-related toxicity was reported, but the included trials were underpowered to detect rare local anesthetic systemic toxicity events and did not consistently capture subclinical neurologic symptoms such as perioral numbness or visual disturbance. **Conclusions:** in adult open abdominal surgery, perioperative IVLI may provide opioid-sparing and recovery benefits, particularly when infusion continues beyond the intraoperative period. However, the certainty of evidence remains limited.

## 1. Introduction

Major open abdominal surgery continues to represent a high-burden perioperative setting despite the widespread adoption of minimally invasive techniques and the development of fast-track perioperative pathways [1,2,3,4,5,6]. Open colorectal resection, gastrectomy, hepatectomy, pancreatic surgery, radical retropubic prostatectomy, and complex mixed abdominal operations remain necessary for advanced pathology, hostile anatomy, oncological requirements, adhesions, conversion from minimally invasive surgery, or surgeon- and patient-specific factors [7,8,9]. The open approach produces substantial somatic and visceral nociception, peritoneal and bowel manipulation, neuroendocrine stress activation, and a systemic inflammatory response that can delay functional recovery [6,8,10].

Postoperative pain after laparotomy is not merely a subjective symptom; it contributes to sympathetic activation, shallow breathing, impaired coughing, delayed mobilization, sleep disruption, and higher opioid exposure [6,10]. Opioids remain effective for rescue analgesia but worsen nausea, vomiting, sedation, urinary retention, and gastrointestinal dysmotility. In abdominal surgery, this creates a clinically important loop: more pain leads to more opioids, and more opioids amplify postoperative ileus, which in turn prolongs hospitalization and delays oral intake. Postoperative ileus itself is multifactorial, involving autonomic inhibition, bowel wall inflammation, fluid imbalance, electrolyte shifts, and opioid-mediated suppression of enteric neural activity [7,8,9].

Enhanced Recovery After Surgery (ERAS) pathways seek to interrupt this loop through multimodal analgesia, avoidance of excessive perioperative fluid administration, early mobilization, early feeding, and prevention of unnecessary nasogastric decompression [6,10]. However, the optimal non-opioid analgesic regimen for major open abdominal procedures is not settled. Epidural analgesia is effective in selected open abdominal procedures but may be contraindicated, technically unsuccessful, or undesirable in patients with coagulopathy, infection risk, anticoagulant therapy, hypotension, or patient refusal. As a result, clinicians continue to search for systemic adjuncts that reduce opioid exposure without adding meaningful procedural risk [10,11,12,13].

Systemic lidocaine is one such adjunct. At perioperative infusion concentrations, lidocaine blocks voltage-gated sodium channels but also modulates central sensitization, suppresses ectopic neuronal discharge, attenuates inflammatory cell activation, and may reduce cytokine-mediated bowel dysfunction [11,12,13]. These mechanisms are particularly relevant after open abdominal surgery, where tissue trauma and bowel manipulation produce both nociceptive and inflammatory recovery barriers [8,11,12]. The pharmacological appeal of IVLI is therefore broader than analgesia alone: it may reduce opioid use, pain during mobilization, ileus duration, nausea, and hospital stay [11,13,14,15,16,17,18].

Safety considerations are also mechanistically important. Lidocaine toxicity is primarily related to excessive free plasma concentrations that block voltage-gated sodium channels in the central nervous system and myocardium. Early neurologic manifestations may include circumoral or perioral numbness, metallic taste, tinnitus, dizziness, visual disturbance, agitation, or tremor, while more severe toxicity can progress to seizures, conduction delay, ventricular arrhythmias, hypotension, or cardiovascular collapse. These pathophysiological risks justify careful patient selection, weight-adjusted dosing, infusion-pump safeguards, electrocardiographic and clinical monitoring, and immediate access to lipid emulsion rescue when IVLI is used [11,13].

Previous reviews have generally supported a possible benefit of IVLI but have frequently pooled laparoscopic, robotic, open abdominal, and non-abdominal operations [14,15,16,17,18]. This creates important clinical heterogeneity. Minimally invasive procedures often have lower tissue trauma, faster baseline recovery, shorter length of stay, and a narrower margin for improvement. Large trials in minimally invasive colorectal surgery have also shown neutral results, reinforcing the need to interpret open surgery separately from laparoscopic or robotic cohorts [19]. Accordingly, this review focuses on randomized evidence in adult major open abdominal surgery and follows PRISMA 2020 reporting standards [1,2].

## 2. Materials and Methods

### 2.1. Review Design and Reporting Standard

This systematic review was conducted and reported according to the PRISMA 2020 statement [1] and the PRISMA-S extension for search reporting [2]. The review question followed the PICOS framework. Population: adults aged 18 years or older undergoing elective major open abdominal surgery through laparotomy or open retropubic access. Intervention: perioperative systemic intravenous lidocaine infusion, with or without a loading bolus, initiated before incision, at induction, or during the intraoperative period, and continued intraoperatively with or without postoperative continuation. Comparator: placebo, standard care, or active analgesic comparator such as thoracic epidural analgesia. Outcomes: postoperative opioid consumption, pain scores, gastrointestinal recovery, length of hospital stay, postoperative nausea and vomiting, inflammatory or stress biomarkers, and adverse events. Study design: randomized controlled trials, including superiority and noninferiority trials [1,2,3,4,5].

A protocol was prepared before study selection. The protocol was not prospectively registered. To resolve the apparent inconsistency noted during peer review, the OSF record (https://osf.io/3uwst) is now described as a retrospective transparency record created after study selection and data extraction, not as prospective registration. No eligibility-criterion amendments were made after final study selection. No amendments were made to the eligibility criteria after final study selection, except that active-comparator randomized trials were retained in a distinct synthesis stratum because they directly inform the clinical role of IVLI when epidural analgesia is the competing strategy [20,21,22,23,24,25,26,27].

### 2.2. Information Sources and Search Strategy

The search strategy covered PubMed/MEDLINE, Embase, Cochrane Central Register of Controlled Trials (CENTRAL), Web of Science Core Collection, Scopus, ClinicalTrials.gov, and the WHO International Clinical Trials Registry Platform, consistent with PRISMA 2020 and PRISMA-S reporting principles [1,2]. The final searches were run and exported on 15 January 2026. Searches covered database inception to 15 January 2026; no lower publication-date limit was applied, which allowed older open-surgery trials, including studies published in 1985, to be captured. No language restriction was applied at the search stage. Human/adult and randomized/interventional limits were applied only when they were native to the database or registry interface and did not remove potentially relevant randomized trials. Trial-register searches were conducted in ClinicalTrials.gov and WHO ICTRP using lidocaine/lignocaine plus abdominal surgery/laparotomy/colorectal-surgery concepts, with interventional/randomized filters applied when available and trial status not used as an exclusion filter. Reference lists of included randomized trials and prior systematic reviews were manually screened, and forward citation tracking was performed for the major included studies [14,15,16,17,18,19,20,21,22,23,24,25,26,27,28,29]. Search concepts combined terms for lidocaine/lignocaine, systemic or intravenous infusion, abdominal or colorectal surgery, laparotomy/open procedures, and randomized trial design. The detailed database-specific strategies are provided in Appendix A.

The search log identified 870 records: 847 from electronic databases and 23 from registers, reference lists, or citation chasing. After duplicate removal, 614 records were screened. These counts are used consistently in the PRISMA flow diagram and checklist package accompanying this manuscript [1,2]. Appendix A now reports the final source-specific strategies as searches that were run and exported for submission, replacing the previous wording that suggested searches were still to be performed.

### 2.3. Eligibility Criteria

For this review, major open abdominal surgery was operationalized as elective surgery performed through laparotomy or an open retropubic abdominal/pelvic access route, with expected inpatient recovery and clinically relevant postoperative pain, opioid exposure, and gastrointestinal or functional recovery endpoints. Open cholecystectomy and radical retropubic prostatectomy were retained because the included randomized trials used open incisions and evaluated the same systemic recovery domains as open colorectal and mixed abdominal procedures. Because these procedures remain clinically heterogeneous, we did not assume exchangeability for meta-analysis; instead, procedure type and comparator type were explicitly described, and placebo/usual-care trials were interpreted separately from active epidural-comparator trials (Table 1).

### 2.4. Study Selection and Data Extraction

Two reviewers independently screened titles/abstracts and then full-text reports, consistent with systematic-review conduct recommended in PRISMA and Cochrane guidance [1,3]. Disagreements were resolved through consensus and, when necessary, third-reviewer adjudication. Third-reviewer adjudication was required for three full-text reports, mainly for unclear surgical approach, comparator classification, or route of lidocaine administration; all remaining discrepancies were resolved by consensus between the two primary reviewers. Data extraction was also performed independently by two reviewers using a prespecified extraction form and was reconciled before synthesis. Extracted variables included publication year, country, surgical procedure, randomized sample size, comparator type, lidocaine loading dose, infusion rate, infusion duration, postoperative continuation, background analgesia, opioid conversion method, pain scale and time points, gastrointestinal recovery definitions, length of stay, postoperative nausea and vomiting, adverse events, and inflammatory or neuroendocrine biomarkers. When outcomes were reported in multiple formats, prespecified clinically interpretable data were prioritized over exploratory time points [3].

Opioid outcomes were preferentially abstracted as intravenous morphine equivalents. When conversion was required, standard equianalgesic assumptions were applied and described in the data table. Median and interquartile range data were not converted unless the source provided sufficient information for transparent transformation [30]. Because several older studies presented outcomes graphically or incompletely, the synthesis emphasized direct extractable values and direction-of-effect coding rather than forcing inappropriate quantitative pooling [3,30].

### 2.5. Risk of Bias, Certainty, and Synthesis Methods

Risk of bias was assessed using the Cochrane RoB 2 tool [3,4] across the randomization process, deviations from intended interventions, missing outcome data, outcome measurement, and selective reporting. Each domain was classified as low risk, some concerns, or high risk, followed by an overall judgment. Certainty of evidence was summarized using GRADE concepts [5] across risk of bias, inconsistency, indirectness, imprecision, and publication bias. GRADE ratings were deliberately conservative and were downgraded when small sample sizes, heterogeneous procedures, active comparators, inconsistent outcome definitions, or incomplete adverse-event reporting limited confidence in the effect estimate. Because the included trials varied substantially in surgical indication, comparator, infusion duration, recovery definitions, and outcome reporting, the primary synthesis was narrative and tabular [3,4,5,20,21,22,23,24,25,26,27,28,29]. Visual synthesis included a PRISMA flow diagram, sample-size chronology, risk-of-bias matrix, all-trial key outcome summary, direction-of-effect heatmap, and dosing schematic [1,3,4]. Meta-analysis was not performed when fewer than three clinically homogeneous studies reported comparable summary statistics for the same outcome at the same time point [3]. A retrospective OSF record was made available at https://osf.io/3uwst to improve transparency; it is not presented as prospective registration.

## 3. Results

### 3.1. Study Selection

**Figure 1:**
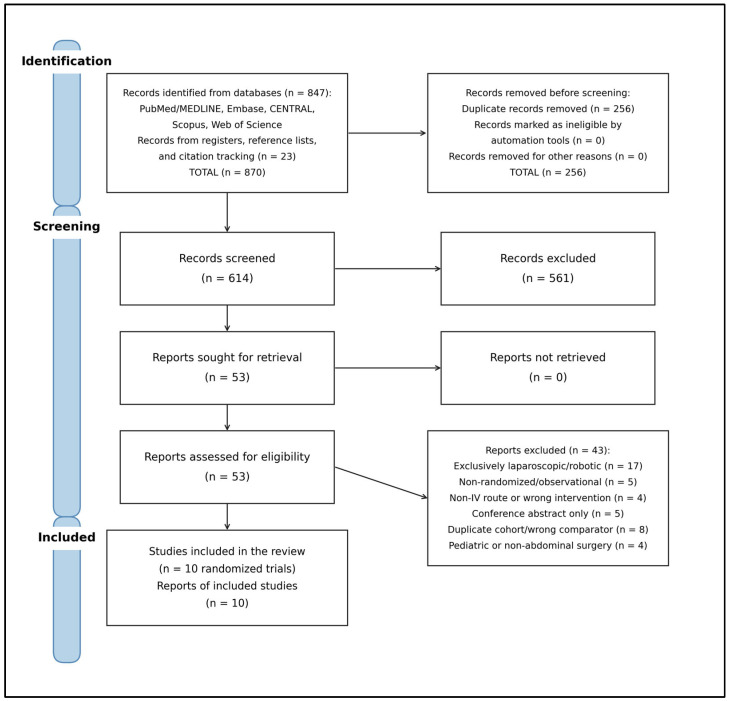
PRISMA 2020 flow diagram [1].

The finalized selection process is shown in Figure 1. After applying the eligibility criteria and screening methods described above, ten randomized trials involving 658 randomized participants were included in the final qualitative synthesis [20,21,22,23,24,25,26,27,28,29]. Detailed record counts and full-text exclusion categories are reported in Section 2.2 and Appendix B to avoid duplicating methodological flow information in the Results.

### 3.2. Characteristics of Included Studies

**Figure 2:**
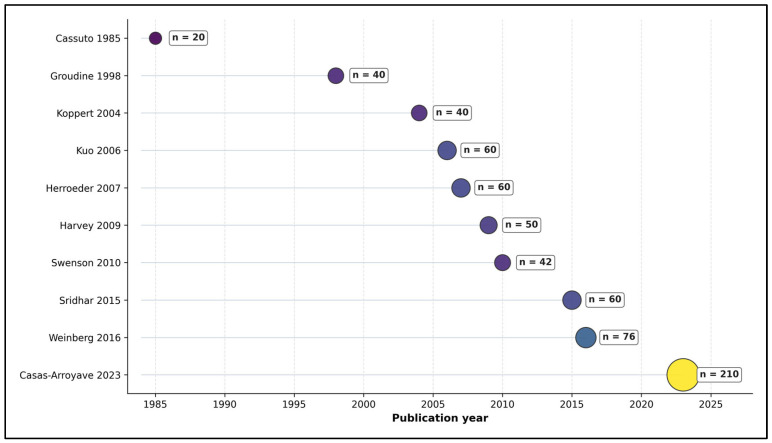
Chronology and sample-size distribution of the included randomized trials [20,21,22,23,24,25,26,27,28,29].

The 10 included trials were published between 1985 and 2023 and enrolled 20 to 210 participants each [20,21,22,23,24,25,26,27,28,29]. Surgical populations included open cholecystectomy, radical retropubic prostatectomy, open colonic or colorectal resection, and mixed major abdominal surgery [20,21,22,23,24,25,26,27,28,29]. Eight trials used saline/placebo or usual-care controls, whereas two trials directly compared IVLI with epidural analgesia [20,21,22,23,24,25,26,27,28,29]. Infusion duration ranged from intraoperative-only protocols with brief postoperative continuation to 24 h postoperative infusion [20,21,22,23,24,25,26,27,28,29]. Five trials included a loading bolus of approximately 1.5 to 2 mg/kg, whereas others used fixed-rate infusions such as 2 to 3 mg/min [21,22,23,24,25,26,27,28,29]. For interpretation, the eight placebo/usual-care trials were treated as evidence for additive efficacy against standard care, whereas the two active-comparator trials were treated as comparative-effectiveness evidence against epidural analgesia and were not numerically merged with placebo-controlled trials (Table 2 and Figure 2).

### 3.3. Risk of Bias

Overall risk of bias was low in three trials, while seven trials raised some concerns according to Cochrane RoB 2 domains [3,4,20,21,22,23,24,25,26,27,28,29]. The most common concerns were unclear allocation concealment in older trials, limited information on prespecified outcomes, incomplete blinding feasibility in active-comparator studies, and small sample sizes that increased imprecision [3,4,20,21,22,23,24,25,26,27,28,29]. No included study was judged to have definitive high risk of bias across the overall assessment.

**Figure 3:**
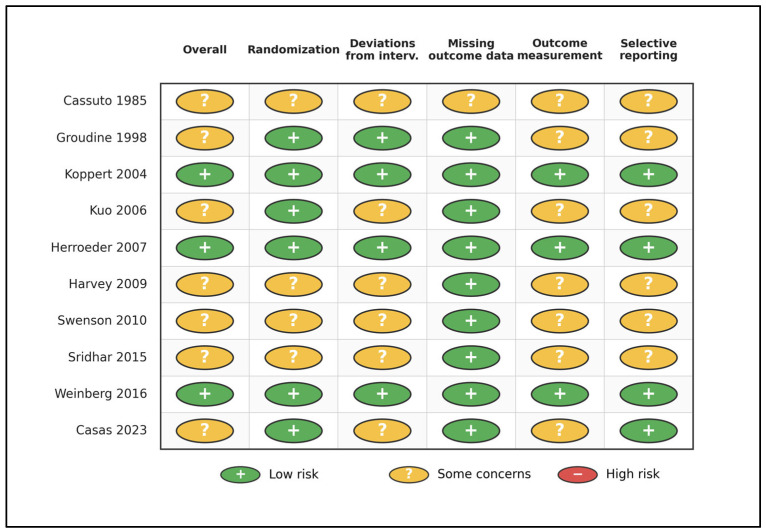
Risk-of-bias summary using Cochrane RoB 2 domains [3,4].

The older trials most often generated “some concerns” because allocation concealment, protocol availability, or prespecified outcome reporting were incompletely described by contemporary standards. Active-comparator trials also raised blinding-related concerns because epidural analgesia is procedurally difficult to mask. Conversely, missing outcome data were generally limited, the main clinical outcomes were prespecified or clinically standard, and no trial showed sufficient domain-level problems to justify an overall high-risk judgment. These explanations were added to make the RoB 2 judgments more transparent rather than to imply that older trials met current reporting standards (Figure 3).

### 3.4. Primary Outcomes: Opioid Consumption and Pain Intensity

Opioid consumption generally favored IVLI in placebo-controlled trials [21,22,23,24,25,26,28,29]. Koppert et al. reported 72 h morphine consumption of 103.1 mg in the lidocaine group versus 159.0 mg in the saline group, corresponding to an absolute reduction of 55.9 mg [23]. Weinberg et al. reported a 24 h morphine reduction of 13.9 mg compared with saline [29]. Other trials described lower patient-controlled analgesia or rescue opioid use but did not always provide extractable summary statistics [21,22,24,26,28]. In active-comparator evidence, Swenson et al. found broadly comparable analgesic recovery to epidural bupivacaine, while Casas-Arroyave et al. reported a prespecified noninferiority result for early dynamic pain when IVLI was compared with thoracic epidural analgesia at 24 h, with only a small difference in early morphine consumption [20,27]. These active-comparator results were interpreted separately from placebo-controlled superiority trials because they address whether IVLI can approximate epidural analgesia, not whether IVLI is superior to no lidocaine.

**Figure 4:**
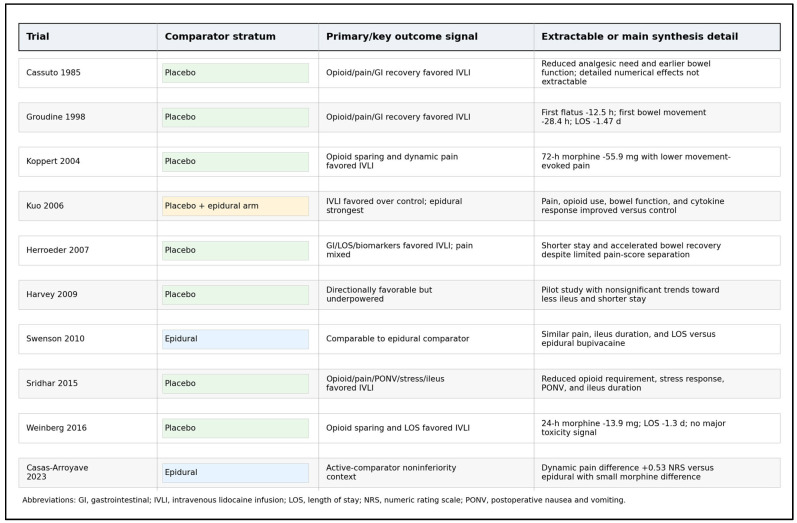
All-trial key outcome summary including all 10 randomized trials [20,21,22,23,24,25,26,27,28,29]. Quantitative effects are shown when extractable; otherwise, the primary direction or active-comparator interpretation is summarized. Active-comparator findings are interpreted separately from placebo-controlled trials.

Pain intensity outcomes were more heterogeneous than opioid outcomes [20,21,22,23,24,25,26,27,28,29]. Some trials reported lower pain at rest or movement, whereas Herroeder et al. found improved bowel recovery and inflammatory marker attenuation without a significant pain-score difference [21,22,23,24,25,26,28,29]. This dissociation supports the possibility that IVLI benefits recovery partly through anti-inflammatory or prokinetic pathways rather than direct analgesia alone [11,12,25]. Pain outcomes were also difficult to compare because scales, postoperative time points, rest-versus-movement conditions, and background analgesic regimens varied substantially across studies [20,21,22,23,24,25,26,27,28,29] (Table 3 and Figure 4).

### 3.5. Secondary Outcomes: Gastrointestinal Recovery, Length of Stay, PONV, and Biomarkers

Gastrointestinal recovery outcomes were the most consistently favorable secondary outcomes in placebo-controlled open-surgery trials [21,22,23,24,25,26,28,29]. Groudine et al. reported first flatus 12.5 h earlier and first bowel movement 28.4 h earlier with IVLI [22]. Herroeder et al. and Sridhar et al. also reported accelerated bowel recovery, while Harvey et al. showed directionally favorable but nonsignificant findings in a pilot sample [25,26,28]. The active-comparator trial by Swenson et al. suggested that IVLI may approximate epidural analgesia for ileus duration and length of stay in open colon resection [27].

Length of stay was variably reported but favored IVLI in the trials with the clearest extractable estimates [22,25,29]. Groudine et al. reported a reduction from 5.22 to 3.75 days, and Weinberg et al. reported a mean reduction of 1.3 days [22,29]. Herroeder et al. reported an approximately one-day reduction after colorectal surgery [25]. PONV was less frequently reported as a primary endpoint, but available data suggested fewer nausea/vomiting events in some trials, likely mediated by opioid sparing [23,28,29]. Biomarker studies provided mechanistic support: Herroeder et al. found attenuation of IL-6, IL-8, complement C3a, CD11b expression, and platelet-leukocyte aggregation, while Kuo et al. and Sridhar et al. reported attenuation of inflammatory or stress responses [24,25,28] (Table 4).

**Figure 5:**
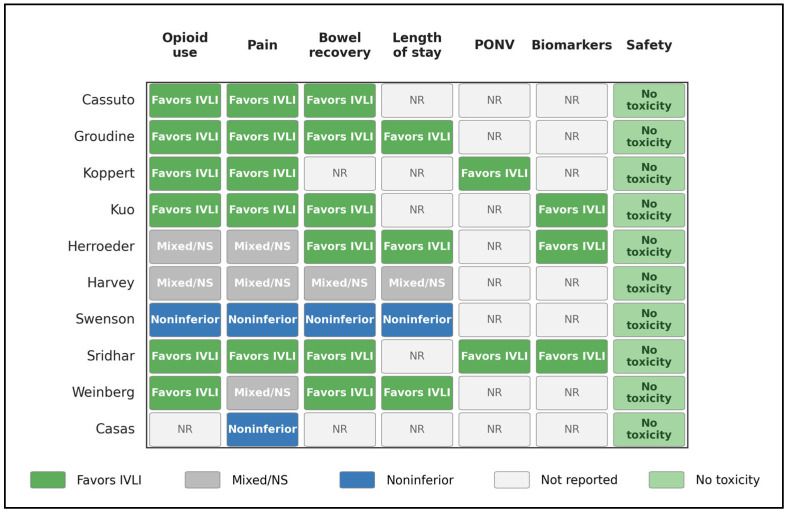
Direction-of-effect map across prespecified outcomes [20,21,22,23,24,25,26,27,28,29]. IVLI, intravenous lidocaine infusion; NR, not reported; NS, not statistically significant; PONV, postoperative nausea and vomiting.

Subclinical local anesthetic systemic toxicity monitoring was inconsistently reported. Most trials stated that no serious lidocaine toxicity occurred, but only some described plasma lidocaine measurement or detailed clinical surveillance. Symptoms such as perioral numbness, metallic taste, tinnitus, dizziness, visual disturbance, tremor, or early neurocognitive changes were not uniformly captured as prespecified safety outcomes. Therefore, the absence of reported serious toxicity should be interpreted as reassuring but not definitive evidence of rare-event safety (Figure 5).

### 3.6. Dosing Patterns and Practical Implementation

**Figure 6:**
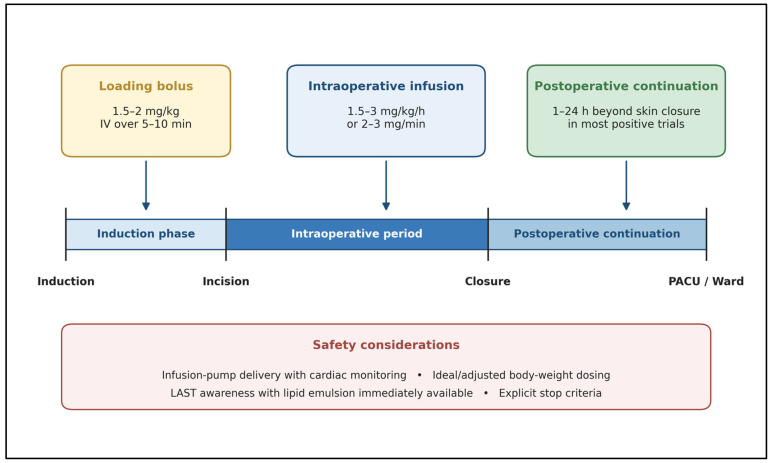
Practical dosing schematic derived from included perioperative protocols [20,21,22,23,24,25,26,27,28,29]. This figure summarizes study protocols and is not a standalone prescribing recommendation.

The most common positive protocols combined a loading bolus of 1.5 to 2 mg/kg with a continuous infusion of 1.5 to 3 mg/kg/h or 2 to 3 mg/min [23,24,25,26,28,29]. Postoperative continuation appeared clinically relevant: trials with continuation beyond skin closure more consistently reported recovery benefits than protocols limited to intraoperative exposure alone [22,25,26,28,29]. However, dosing was not standardized and some studies used fixed mg/min rates, while others used weight-based dosing [20,21,22,23,24,25,26,27,28,29]. The optimal dose, body-weight scalar, and postoperative duration therefore remain unresolved [11,13] (Figure 6). Summary of evidence is presented in Table 5.

## 4. Discussion

### 4.1. Principal Findings

This systematic review found that perioperative IVLI may be associated with opioid-sparing and selected recovery benefits in adult patients undergoing major open abdominal surgery, but the certainty of evidence remains limited [20,21,22,23,24,25,26,27,28,29]. Compared with placebo or usual care, IVLI generally reduced opioid consumption, accelerated gastrointestinal recovery, and shortened length of stay in several trials [21,22,23,24,25,26,28,29]. The largest active-comparator trial suggested comparable early dynamic pain outcomes versus thoracic epidural analgesia within its noninferiority design after open major abdominal surgery, but this should be interpreted separately from placebo-controlled evidence because epidural analgesia is an active and highly effective comparator [20].

The results are biologically plausible. Open abdominal surgery produces tissue injury, visceral manipulation, inflammatory activation, and postoperative neural sensitization [6,8,10]. Lidocaine can plausibly address several of these pathways through sodium-channel blockade, attenuation of ectopic neuronal activity, reduction of hyperalgesia, and inflammatory modulation [11,12,13]. Improved gastrointestinal recovery is likely multifactorial rather than attributable to a single prokinetic effect. IVLI may indirectly accelerate bowel recovery by reducing opioid exposure and thereby limiting opioid-mediated suppression of enteric neural activity. It may also have direct anti-inflammatory and neuroimmune effects within the postoperative ileus pathway, including attenuation of cytokine release, leukocyte activation, and visceral afferent sensitization after bowel manipulation [8,11,12,13,25]. This broad mechanism may explain why some studies showed improved bowel recovery without strong pain-score separation [24,25,28]. In other words, IVLI should be viewed as a recovery adjunct, not merely as an analgesic drug [11,13].

The available evidence also clarifies why open and minimally invasive procedures should not be pooled without caution [14,15,16,17,18,19]. The large ALLEGRO randomized trial in elective minimally invasive colonic surgery reported no improvement in return of gut function with IV lidocaine, despite earlier smaller trials suggesting benefit [19]. This does not automatically negate the open-surgery signal because the magnitude and mechanisms of surgical stress differ [6,8,10]. However, it does raise the bar for future open-surgery trials: small single-center studies with variable endpoints are no longer sufficient to establish routine practice [3,5,20,21,22,23,24,25,26,27,28,29].

### 4.2. Clinical Implications

For clinical practice, IVLI may be most reasonable when the expected pain burden and ileus risk are high, when opioid-sparing is a priority, and when epidural analgesia is contraindicated, declined, unavailable, or technically unsuccessful [10,11,13,20,21,22,23,24,25,26,27,28,29]. Potentially favorable patient-related factors include high baseline risk of ileus, anticipated high opioid requirement, intolerance of neuraxial techniques, anticoagulant use that precludes epidural placement, or a need to avoid excessive sedation and opioid-related nausea. The findings support consideration of IVLI within structured ERAS pathways for selected open abdominal procedures, particularly when infusion can be delivered safely by pump with cardiac monitoring and clear local anesthetic systemic toxicity protocols [10,11,13].

The evidence does not support indiscriminate use. Patients with significant conduction disease, severe hepatic dysfunction, seizure risk, concomitant class I antiarrhythmics, severe frailty with altered protein binding, or inability to receive appropriate monitoring require careful risk-benefit assessment [11,13]. Older age, hypoalbuminemia, low lean body mass, hepatic impairment, heart failure, interacting antiarrhythmics, and neurologic vulnerability may increase the free lidocaine fraction or reduce physiologic reserve, thereby lowering the margin between therapeutic and toxic concentrations. The relevant adverse-effect pathway is concentration-dependent sodium-channel blockade in the central nervous system and myocardium, which explains why early neurologic symptoms can precede seizures or cardiovascular instability. Institutions adopting IVLI should use standardized dosing, ideal or adjusted body-weight rules where appropriate, maximum infusion rates, handoff checklists, rescue lipid emulsion availability, and explicit stop criteria [11,13].

### 4.3. Methodological Interpretation

The review intentionally avoided a forced meta-analysis of heterogeneous outcomes [3]. Several included trials reported opioid consumption using different time windows, pain scores at different postoperative conditions, and bowel recovery using inconsistent definitions [20,21,22,23,24,25,26,27,28,29]. A pooled estimate under these conditions could appear statistically precise while being clinically misleading. Direction-of-effect and extractable-effect graphics were therefore used to preserve interpretability [3,5].

Active-comparator studies answer a different question than placebo-controlled trials. Placebo-controlled trials estimate whether IVLI adds benefit to standard care [21,22,23,24,25,26,28,29]. Active-comparator trials estimate whether IVLI can replace or approximate epidural analgesia [20,27]. The latter question is clinically important but should not be merged numerically with placebo-controlled superiority trials without careful transitivity assumptions [3,5].

### 4.4. Limitations

This review has several limitations. First, most included trials were small, and several were performed before contemporary ERAS pathways, limiting certainty and external validity [5,10,21,22,23,24,25,26,27,28,29]. Second, although the final searches were run and exported on 15 January 2026, the submission package should retain the complete exported search files and deduplication log for auditability [1,2]. Third, most studies did not consistently report plasma lidocaine levels, rare toxicity outcomes, or protocol deviations [20,21,22,23,24,25,26,27,28,29]. Fourth, outcome definitions were heterogeneous, particularly for postoperative ileus and return of gastrointestinal function [7,20,21,22,23,24,25,26,27,28,29]. Fifth, the review focused on open abdominal surgery and therefore excluded large contemporary minimally invasive trials from the included-study pool, although these trials were discussed as contextual evidence [19].

Finally, the certainty of safety evidence remains limited [5]. The absence of reported serious toxicity in 658 randomized participants is reassuring but insufficient to exclude uncommon local anesthetic systemic toxicity, arrhythmia, or medication administration errors [20,21,22,23,24,25,26,27,28,29]. Large pragmatic trials and registry-linked safety analyses are needed to define rare adverse events and identify high-risk subgroups [11,13].

### 4.5. Future Research

Future trials should focus on contemporary open abdominal populations, use standardized IVLI dosing, weight-scaling rules, maximum infusion limits, monitoring procedures, and explicit LAST stop/rescue algorithms, prespecify gastrointestinal recovery endpoints such as GI-2 or GI-3, report opioid consumption in morphine equivalents at standardized time points, and include patient-centered recovery outcomes such as quality of recovery, mobilization, sleep, and persistent opioid use [7,11,13,30]. Future protocols should also state whether subclinical toxicity symptoms are actively assessed and should report plasma lidocaine levels or predefined safety thresholds when feasible. Comparative-effectiveness studies should stratify epidural-eligible versus epidural-ineligible patients and report technical epidural failure rates [20,27]. Economic analyses are also warranted because even a one-day reduction in length of stay could be meaningful if the intervention is safe and low cost [9,22,25,29]. Nevertheless, these findings should be interpreted in light of potential residual confounding from unmeasured or incompletely controlled factors, including underlying comorbidities and other patient- and treatment-related characteristics [31,32,33,34,35].

## 5. Conclusions

Perioperative intravenous lidocaine infusion is a biologically plausible adjunct for selected adult patients undergoing major open abdominal surgery, but the current randomized evidence should be interpreted as suggestive rather than definitive. The randomized evidence suggests opioid-sparing effects, faster gastrointestinal recovery, and possible length-of-stay reductions compared with placebo or usual care, with active-comparator data suggesting that IVLI may produce comparable early dynamic pain outcomes to epidural analgesia in selected settings. Nevertheless, certainty remains limited by small trials, heterogeneity, and incomplete toxicity reporting. The lack of reported serious lidocaine toxicity is reassuring but does not exclude rare adverse events or unreported subclinical local anesthetic systemic toxicity symptoms. IVLI should be implemented only within protocolized ERAS pathways with clear monitoring and safety procedures, while future open-surgery-specific randomized trials should define the optimal dose, duration, patient selection, and recovery endpoints.

## Figures and Tables

**Table 1:** Eligibility framework for the systematic review, structured according to PICOS and PRISMA 2020 reporting principles [1].

Domain	Inclusion Criteria	Exclusion Criteria
Population	Adults undergoing elective major open abdominal surgery, including open colorectal surgery, open cholecystectomy, radical retropubic prostatectomy, and mixed major abdominal procedures.	Pediatric participants; non-abdominal operations; exclusively laparoscopic, robotic, endoscopic, or minor abdominal procedures.
Intervention	Systemic perioperative intravenous lidocaine/lignocaine infusion, with or without loading bolus and with any intraoperative or postoperative duration.	Epidural, intrathecal, wound infiltration, topical, intraperitoneal-only, or oral lidocaine without systemic IV infusion.
Comparator	Placebo/saline, usual care without IV lidocaine, or active analgesic comparator such as thoracic epidural analgesia.	No comparator; non-randomized historical controls; duplicate secondary reports without unique clinical outcomes.
Outcomes	At least one predefined clinical outcome: opioid consumption, pain intensity, bowel function, postoperative ileus, length of stay, PONV, inflammatory markers, or safety.	Pharmacokinetic-only studies without clinical outcomes; conference abstracts without usable full text.
Design	Randomized controlled superiority or noninferiority trials.	Observational designs, reviews, letters, animal studies, case reports, and protocols without results.

**Table 2:** Characteristics of included randomized trials [20,21,22,23,24,25,26,27,28,29].

Study	Participants/Surgery	*n*	IV Lidocaine Protocol	Comparator	Main Reported Findings
Cassuto et al., 1985 [21]	Open cholecystectomy; Sweden	20	Continuous low-dose IV infusion; intraoperative and postoperative exposure	Saline	Reduced analgesic requirements and earlier bowel function; no serious toxicity reported.
Groudine et al., 1998 [22]	Open radical retropubic prostatectomy; USA	40	2 mg/min from induction to 1 h after surgery	Saline	Earlier flatus (18.9 vs. 31.4 h), earlier bowel movement (38.8 vs. 67.2 h), lower pain, and shorter stay (3.75 vs. 5.22 d).
Koppert et al., 2004 [23]	Major open abdominal surgery; Germany	40	1.5 mg/kg bolus then 1.5 mg/kg/h until 1 h postoperatively	Saline	Lower 72 h morphine use (103.1 vs. 159.0 mg) and less pain on movement.
Kuo et al., 2006 [24]	Open colonic surgery; Taiwan	60	2 mg/kg bolus then 3 mg/kg/h infusion	Saline and thoracic epidural arm	IVLI improved pain, opioid use, and bowel function compared with control; epidural arm often had strongest analgesic effect.
Herroeder et al., 2007 [25]	Open colorectal surgery; Germany	60	1.5 mg/kg bolus then 2 mg/min until 4 h postoperatively	Saline	Shorter hospital stay, accelerated bowel recovery, and attenuated IL-6, IL-8, C3a, CD11b, and platelet-leukocyte activation; pain scores similar.
Harvey et al., 2009 [26]	Open bowel resection; Canada	50	1.5 mg/kg bolus then 3 mg/min until 24 h postoperatively	Saline	Pilot trial showing directionally favorable but underpowered effects on ileus and length of stay.
Swenson et al., 2010 [27]	Open colon resection; USA	42	2 mg/min without bolus; continued for 24 h postoperatively	Epidural bupivacaine	IVLI had comparable pain, ileus duration, and length of stay to epidural bupivacaine.
Sridhar et al., 2015 [28]	Elective open abdominal surgery; India	60	1.5 mg/kg bolus then 1.5 mg/kg/h until 8 h postoperatively	Saline	Reduced opioid requirements, PONV, stress response, and postoperative ileus.
Weinberg et al., 2016 [29]	Open radical retropubic prostatectomy; Australia	76	Preoperative 0.075 mL/kg 2% lidocaine bolus, intraoperative IV infusion, and 24 h postoperative subcutaneous infusion	Saline	Reduced 24 h morphine consumption by 13.9 mg and shortened stay by 1.3 days; no major safety signal.
Casas-Arroyave et al., 2023 [20]	Open major abdominal surgery; Colombia	210	Systemic IV lidocaine protocol compared with thoracic epidural analgesia	Thoracic epidural bupivacaine with morphine	Noninferior dynamic pain at 24 h; 24 h morphine difference 1.8 mg; no difference in adverse events or procedural complications.

**Table 3:** Primary outcome synthesis [20,21,22,23,24,25,26,27,28,29].

Study	Opioid Outcome	Pain Outcome	Interpretation	Extractable Effect
Cassuto 1985 [21]	Reduced analgesic requirement reported.	Lower postoperative pain scores.	Favors IVLI, but reporting limited.	Not extractable.
Groudine 1998 [22]	Lower PCA morphine shown graphically.	Lower VAS at early time points.	Favors IVLI.	GI recovery and LOS extractable.
Koppert 2004 [23]	72 h morphine 103.1 vs. 159.0 mg.	Less pain on movement; rest pain low in both groups.	Favors IVLI for opioid sparing and dynamic pain.	−55.9 mg morphine over 72 h.
Kuo 2006 [24]	Lower opioid use than control.	Lower VAS than control at multiple time points.	Favors IVLI versus control; epidural strongest.	Partially extractable.
Herroeder 2007 [25]	PCA morphine similar.	No significant VAS difference.	Clinical recovery benefit without pain-score separation.	No opioid/pain benefit.
Harvey 2009 [26]	Trend toward less opioid use.	Trend toward lower VAS.	Underpowered pilot evidence.	Not extractable.
Swenson 2010 [27]	Comparable to epidural arm.	Comparable to epidural arm.	Active-comparator equivalence signal.	Not directly placebo-comparable.
Sridhar 2015 [28]	Reduced opioid requirement.	Lower postoperative VAS.	Favors IVLI.	Not fully extractable.
Weinberg 2016 [29]	Reduced 24 h morphine use by 13.9 mg.	No clinically important pain difference in corrected abstract; full article reports early rest-pain reduction.	Favors opioid sparing; pain interpretation cautious.	−13.9 mg morphine over 24 h.
Casas 2023 [20]	24 h morphine difference 1.8 mg vs. epidural.	Dynamic pain difference 0.53 points, within noninferiority margin.	Noninferior to thoracic epidural for early dynamic pain.	+0.53 NRS points versus epidural.

GI, gastrointestinal; IVLI, intravenous lidocaine infusion; LOS, length of stay; NRS, numeric rating scale; PCA, patient-controlled analgesia; VAS, visual analog scale.

**Table 4:** Secondary outcomes and safety synthesis [20,21,22,23,24,25,26,27,28,29].

Study	GI Recovery	LOS	PONV	Inflammatory/Stress Response	Safety
Cassuto 1985 [21]	Earlier bowel function.	NR	NR	NR	No serious toxicity; subclinical LAST: NR.
Groudine 1998 [22]	Flatus 18.9 vs. 31.4 h; bowel movement 38.8 vs. 67.2 h.	3.75 vs. 5.22 d.	NR	NR	No serious toxicity; subclinical LAST: NR.
Koppert 2004 [23]	NR	NR	Lower in IVLI group.	NR	No serious toxicity; subclinical LAST: NR.
Kuo 2006 [24]	Earlier bowel function than control.	NR	NR	Reduced cytokine response.	No serious toxicity; subclinical LAST: not uniformly specified.
Herroeder 2007 [25]	Accelerated return of bowel function.	Approximately 1 d shorter.	NR	Reduced IL-6, IL-8, C3a, CD11b, selectins.	Plasma levels below toxic threshold; subclinical LAST: incompletely reported.
Harvey 2009 [26]	Trend earlier; pilot underpowered.	Trend shorter.	NR	NR	No serious toxicity; subclinical LAST: NR.
Swenson 2010 [27]	Comparable to epidural.	Comparable to epidural.	NR	NR	No serious toxicity; subclinical LAST: NR.
Sridhar 2015 [28]	Reduced ileus duration.	NR	Reduced.	Attenuated cortisol and CRP.	No serious toxicity; subclinical LAST: NR.
Weinberg 2016 [29]	Earlier mobilization, oral water and food tolerance.	Reduced by 1.3 d.	No major difference.	NR	Plasma levels measured; no clinically important toxicity signal; subclinical LAST: incompletely reported.
Casas 2023 [20]	NR	NR	NR	NR	Adverse events similar to epidural; subclinical LAST: not uniformly specified.

**Table 5:** Summary certainty of evidence using GRADE concepts [5,20,21,22,23,24,25,26,27,28,29].

Outcome	Main Finding	Certainty	Reasons for Rating	Clinical Interpretation
Opioid consumption	Most placebo-controlled trials favored IVLI; some statistics were not extractable.	Low to moderate	Downgraded for small trials, heterogeneous procedures/protocols, and incomplete reporting.	Potential opioid-sparing adjunct in selected open procedures, not definitive routine-practice evidence.
Pain intensity	Mixed but generally favorable; active-comparator evidence applies separately.	Low	Downgraded for heterogeneous scales/time points, background analgesia, and comparator differences.	Pain benefit is plausible but should not be the sole rationale for IVLI.
GI recovery/ileus	Several open-surgery placebo-controlled trials favored IVLI.	Low to moderate	Downgraded for small samples, variable definitions, and procedure heterogeneity.	Promising ERAS-relevant signal requiring contemporary confirmation.
Length of stay	Favored IVLI in several trials with extractable estimates.	Low	LOS affected by discharge protocols, geography, era, and ERAS maturity.	Promising but indirect and context-dependent.
PONV	Possible reduction in some trials.	Low	Inconsistent reporting and secondary outcome status.	Likely mediated partly by opioid reduction.
Safety	No serious toxicity reported; subclinical LAST symptoms inconsistently captured.	Low	Underpowered for rare toxicity; symptom-level monitoring incompletely reported.	Reassuring short-term signal only; requires protocolized monitoring.

## Data Availability

All data summarized in this review are available in the cited published studies [20,21,22,23,24,25,26,27,28,29]. The extracted evidence tables and PRISMA checklist documents (Supplementary Materials) are provided with the submission package [1,2].

## Supplementary Materials

The following supporting information can be downloaded at: https://www.mdpi.com/article/10.3390/jcm15114068/s1.

## Appendix A. Database Search Strategies

**Table A1:** Reproducible search strategies for the evidence synthesis [1,2].

Database/Source	Final Search Strategy Run/Exported on 15 January 2026 for the Evidence Synthesis [1,2]
PubMed/MEDLINE	(lidocaine[MeSH Terms] OR lidocaine[tiab] OR lignocaine[tiab] OR “intravenous lidocaine”[tiab] OR “lidocaine infusion”[tiab] OR “systemic lidocaine”[tiab]) AND (abdominal surgery[tiab] OR laparotomy[tiab] OR colorectal[tiab] OR colonic[tiab] OR colectomy[tiab] OR gastrectomy[tiab] OR hepatectomy[tiab] OR pancreatectomy[tiab] OR prostatectomy[tiab]) AND (randomized controlled trial[pt] OR randomized[tiab] OR randomised[tiab] OR placebo[tiab] OR trial[tiab])
Embase	(lidocaine/exp OR lidocaine:ti,ab OR lignocaine:ti,ab OR “intravenous lidocaine”:ti,ab OR “lidocaine infusion”:ti,ab) AND (abdominal surgery/exp OR laparotomy/exp OR colorectal surgery/exp OR colectomy/exp OR gastrectomy/exp OR hepatectomy/exp OR pancreatectomy/exp OR prostatectomy/exp OR abdominal:ti,ab OR laparotomy:ti,ab) AND (randomized controlled trial/exp OR random *:ti,ab OR placebo:ti,ab)
Cochrane CENTRAL	(lidocaine OR lignocaine OR “intravenous lidocaine” OR “lidocaine infusion” OR “systemic lidocaine”) AND (abdominal OR laparotomy OR colorectal OR colonic OR colectomy OR gastrectomy OR hepatectomy OR pancreatectomy OR prostatectomy)
Scopus	TITLE-ABS-KEY(lidocaine OR lignocaine OR “intravenous lidocaine” OR “lidocaine infusion” OR “systemic lidocaine”) AND TITLE-ABS-KEY(“abdominal surgery” OR laparotomy OR colorectal OR colonic OR colectomy OR gastrectomy OR hepatectomy OR pancreatectomy OR prostatectomy) AND TITLE-ABS-KEY(random * OR placebo OR trial)
Web of Science Core Collection	TS = (lidocaine OR lignocaine OR “intravenous lidocaine” OR “lidocaine infusion” OR “systemic lidocaine”) AND TS = (“abdominal surgery” OR laparotomy OR colorectal OR colonic OR colectomy OR gastrectomy OR hepatectomy OR pancreatectomy OR prostatectomy) AND TS = (random * OR placebo OR trial)
ClinicalTrials.gov/WHO ICTRP	lidocaine OR lignocaine; abdominal surgery OR colorectal surgery OR laparotomy; searched from registry inception to 15 January 2026; interventional/randomized filters applied when available; no restriction by recruitment status; records screened for adult open abdominal surgery and systemic IV infusion.

## Appendix B. Full-Text Exclusion Categories

**Table A2:** Full-text exclusion categories summarized for PRISMA 2020 reporting [1].

Reason for Exclusion	Number of Reports
Exclusively laparoscopic or robotic procedures	17
Non-randomized or observational design	5
Non-IV lidocaine route or wrong intervention	4
Conference abstract only without usable full text	5
Duplicate cohort, secondary report, or wrong comparator for synthesis question	8
Pediatric or non-abdominal surgery population	4
Total excluded at full-text stage	43
